# Delayed diagnosis and treatment of tuberculosis in HIV+ patients in Mozambique: A cost-effectiveness analysis of screening protocols based on four symptom screening, smear microscopy, urine LAM test and Xpert MTB/RIF

**DOI:** 10.1371/journal.pone.0200523

**Published:** 2018-07-19

**Authors:** S. Orlando, I. Triulzi, F. Ciccacci, I. Palla, L. Palombi, M. C. Marazzi, M. Giuliano, M. Floridia, S. Mancinelli, E. Mutemba, G. Turchetti

**Affiliations:** 1 Department of Biomedicine, University of Tor Vergata, Rome, Italy; 2 Institute of Management, Scuola Superiore Sant' Anna, Pisa, Italy; 3 Department of Human Science, LUMSA University, Rome, Italy; 4 National Center for Global Health, Istituto Superiore di Sanità, Rome, Italy; 5 DREAM programme, Community of Sant’Egidio, Maputo, Mozambique; University of Cape Town, SOUTH AFRICA

## Abstract

**Background:**

Tuberculosis (TB) represents the ninth leading cause of death worldwide. In 2016 are estimated 1.3 million TB deaths among HIV negative people and an additional 374,000 deaths among HIV positive people. In 2016 are estimated 1.4 million new cases of TB in people living with HIV (PLHIV), 74% of whom were living in Africa. In light of these data, the reduction of mortality caused by TB in PLHIV is strongly required specially in low-income countries as Mozambique. According to international guidelines, the initial TB screening in HIV+ patients should be done with the four symptoms screening (4SS: fever, current cough, night sweats and weight loss). The diagnostic test more used in resource-limited countries is smear microscopy (SMEAR). World Health Organization (WHO) recommended Lateral Flow urine LipoArabinoMannan assay (LF-LAM) in immunocompromised patients; in 2010 WHO endorsed the use of Xpert Mycobacterium Tuberculosis/Rifampicin (MTB/RIF) test for rapid TB diagnosis but the assay is not used as screening test in all HIV+ patients irrespectively of symptoms due to cost and logistical barriers. The paper aims to evaluate the cost-effectiveness of three screening protocols: standard (4SS and SMEAR in positive patients to 4SS); MTB/RIF; LF-LAM / MTB/RIF.

**Methods:**

We developed a model to assess the cost-effectiveness of the MTB/RIF protocol versus the common *standard* and LF-LAM / MTB/RIF protocol. The model considered a sample of 1,000 HIV+ antiretroviral treatment naïve patients in Mozambique. We evaluated disability-adjusted life year (DALY) averted for each protocol, cost per DALY, and incremental cost-effectiveness ratio (ICER), over 1-year, assuming a national healthcare system perspective. The model considered the delayed diagnosis as the time elapsed between a false negative test and the diagnosis and treatment of TB. Additional health system organization delay is defined as the time interval between positive test and treatment initiation caused by a delay in the delivery of results due organization of services. We conducted a sensitivity analysis on more relevant variables.

**Results:**

The MTB/RIF protocol was cost-effective as compared to the standard protocol with an ICER of $56.54 per DALY saved. In a cohort of 1,000 patients MTB/RIF and LF-LAM / MTB/RIF protocol generated 1,281 and 1,254 DALY’s saved respectively, with a difference of 174 and 147 DALY respect to the standard protocol. The total cost of MTB/RIF protocol was lower ($92,263) than the standard ($147,226) and the LF-LAM / MTB/RIF ($113,196). Therefore, the cost per DALY saved including new infections due to delayed diagnosis with the standard protocol was $79.06, about 5 fold higher than MTB/RIF and LF-LAM / MTB/RIF protocols. The cost of additional TB infections due to delays in diagnosis plus health system delay seemed the more relevant costs. The low sensibility and sensitivity of the standard protocol led to a high number of false negatives, thus delayed TB diagnoses and treatment lead to the development of newly transmitted TB infections.

**Conclusions:**

Our study shows that the MTB/RIF adoption could lead to an increasing of TB case-finding and a reduction in costs compared with standard and LF-LAM / MTB/RIF protocols.

## Introduction

Tuberculosis (TB) represents the main cause of death in people living with Human Immunodeficiency Virus (PLHIV), with a large disease burden in Mozambique and in other resource-limited countries [1]. Reducing TB-related deaths among PLHIV is an urgent action according to the Joint United Nations Programme on HIV/Acquired Immune Deficiency Syndrome (UNAIDS) and World Health Organization (WHO) [2].

The risk of developing TB co-infection in PLHIV is 26 to 31 times higher in comparison to HIV negative individuals [3]. PLHIV amounted to 11% of all new TB cases in 2015 [4]. Due to co-infection, TB diagnosis is often challenging in PLHIV, especially in resource-limited countries, with a subsequent delay in TB diagnosis and treatment. In 2016, 374,000 people with TB and HIV co-infection died in addition to the 1.3 million deaths from TB alone [1]. Almost 60% of TB cases among PLHIV were not diagnosed or treated, according to UNAIDS in 2015 [5].

An active case finding using four symptoms screening (positive when present one symptom among: fever, current cough, night sweats and weight loss) (4SS) is the recommended strategy to intensify TB case finding among PLHIV [6]. Smear microscopy (SMEAR) is the most commonly utilized TB diagnostic test in resource-limited settings, but it doesn’t detect most of the cases, especially in PLHIV. The test is accurate only half of the time (43% of TB/HIV co-infected patients) [1].

Other diagnostic options include Xpert mycobacterium tuberculosis/rifampicin (MTB/RIF) and lateral flow urine lipoarabinomannan assay (LF-LAM) that consent rapid detection of TB allowing prompt treatment initiation and reducing ongoing transmissions during the infectious period. These two tests are defined as point of care (POC) [7].

MTB/RIF is a unique, rapid, automated nucleic acid amplification test that detects both TB and rifampicin resistance in sputum, within two hours after test initiation, with minimal hands-on technical time. MTB/RIF is more expensive than conventional sputum microscopy but shows higher specificity and sensitivity [8] in detecting TB cases. In 2010, the WHO recommended its use as initial diagnostic test in PLHIV suspected of having TB [1], thus after being screened with 4SS.

The LF-LAM detects lipopolysaccharide, a component of the bacterial cell wall, present in urine, also recommended by the WHO. The test is low-cost, simple, requires no special equipment, yields results in approximately 30 min, and shows high sensitivity in HIV+ patients when CD4 cells count is lower than 200/mm^3^ [9].

The point of care test should be able to be performed on an easily accessible sample and to provide results in a timely manner, allowing a quick treatment turnaround time of a few minutes or hours (in a single clinical encounter), hence avoiding patient loss-to-follow-up [7].

The present study follows a clinical study performed in Mozambique where diagnostic algorithms to detect TB were tested on HIV+ patients initiating antiretroviral therapy (ART) [10].

The aim of the study is to evaluate the cost effectiveness of three screening protocols in risk group population (PLHIV) to improve pulmonary TB early detection.

## Methods

**Fig 1:**
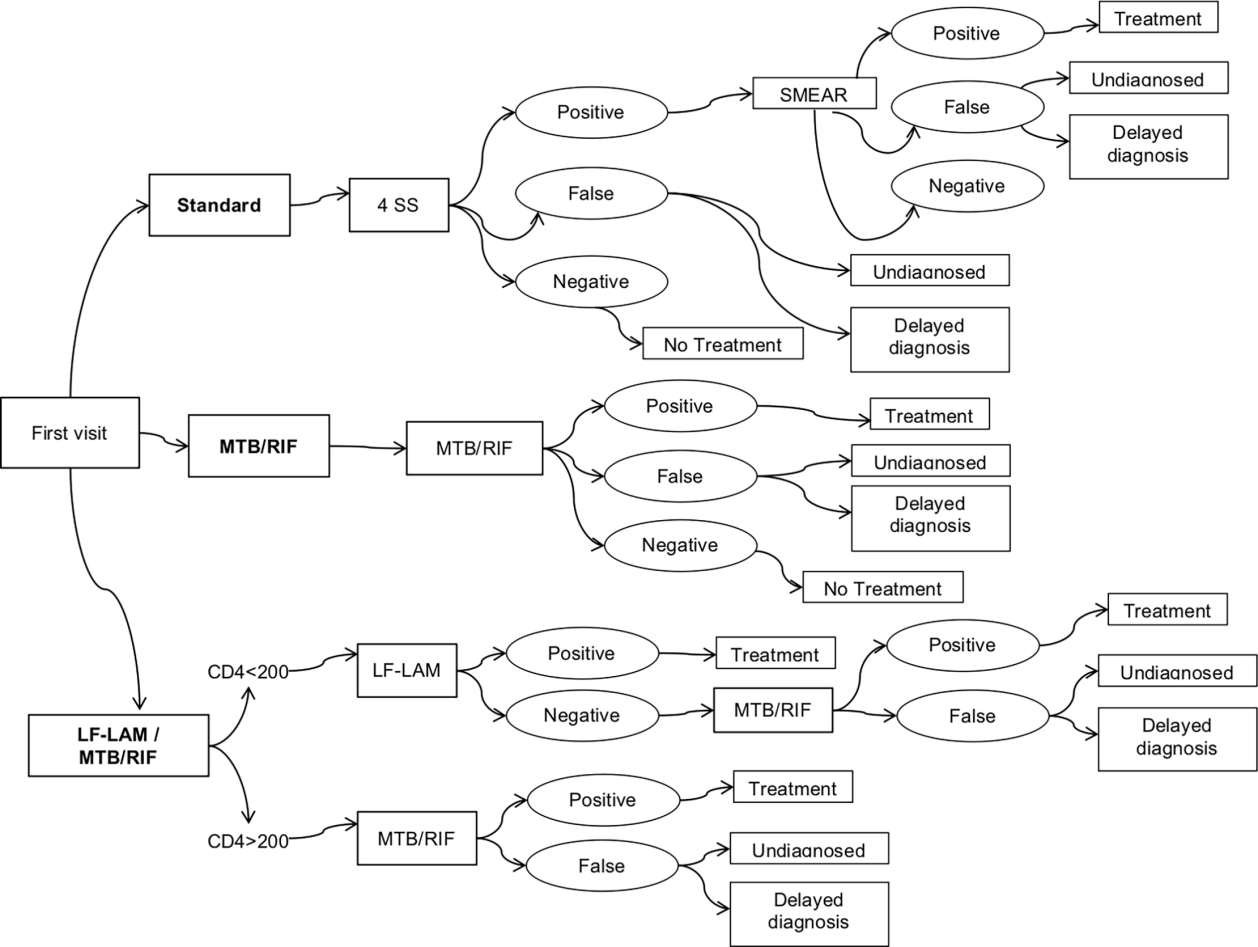
TB screening protocols in HIV+ patients.

This study was conducted in the framework of the “Disease Relief through Excellent and advanced Means” (DREAM) program of the Community of Sant'Egidio in Mozambique. DREAM program has been active in Mozambique since 2002 [11]. Services for TB care were introduced in 2013. The following protocols have been included in the simulation (Fig 1):

1. Standard: 4SS and SMEAR for participants with positive 4SS results.
2. MTB/RIF: MTB/RIF for all participants.
3. LF-LAM / MTB/RIF: LF-LAM in all patients with CD4 cells count <200/mm^3^; MTB/RIF in all patients with CD4 cells count >200/mm^3^ and in those with CD4 cells count <200/mm^3^ and negative LF-LAM results.

Despite being the gold standard test for TB diagnosis, culture test was not included in the present analysis as it is not considered by WHO as an initial diagnostic test due to high resource demand and long time for results delivery [12].

The economic evaluation was performed according to a national healthcare perspective and presented a time horizon of one year.

The model of analysis was performed using Microsoft Excel 2016.

### Effectiveness analysis

In a previous study [10], a TB-diagnosis algorithm based on combined rapid POC assays was evaluated in 972 HIV+ patients in Mozambique. In the present study, to evaluate the cost-effectiveness of the different screening protocols, a simulation was performed on a sample of 1,000 HIV+ patients, with an average age of 35 years at the time of the positive HIV test, as in the previous study. The life expectancy in Mozambique in 2015 of 57.60 years was used [13].

The performance of different protocols is described in Table 1. 4SS was considered positive in the presence of one of the following symptoms: any cough, night sweats, any fever, weight loss. True-positive-, true-negative-, false-positive- and false-negative-cases were derived on the basis of sensitivity and specificity of tests. Sensitivity and specificity of 4SS was considered 77.5% and 70.4% respectively as described by Floridia et al. [10]. This specificity value differed from that reported by the WHO of 50% [1], but data regarding performance of 4SS is widely heterogeneous among studies [14–16]; individual performance assessments for SMEAR, MTB/RIF and LF-LAM were derived from previous studies or international guidelines where all test were compared with gold standard culture tests [17–19].

**Table 1:** Sensitivity and specificity of the screening methods.

Test	Sensitivity (%)	Specificity (%)	Source
*4SS*	77.5	70.4	[10]
*SMEAR*	43.0	100.0	[17]
*MTB/RIF*	97.6	99.2	[18]
*LF-LAM*	49.0	90.0	[19]

The impact of TB treatment on the patients’ health was quantified in terms of Disability-Adjusted Life Years (DALY) saved. No-therapy scenario considered the survival of HIV/TB co-infected patients receiving only ART for HIV, with a range of 0.5 to 0.83 years [20]. Therefore, the average of 0.665 years was considered the base. In the case of patients who were TB screened and treated for both HIV and TB, the considered survival was 12.9 years as described in other studies [20–23]. Disability weights of 0.053 for patient affected by Acquired Immune Deficiency Syndrome (AIDS) receiving ART and 0.399 for patients affected by TB were used to make disability adjustments [21, 24]. We used a standard age-weight function based on that employed by the World Bank [25].

The study assumes that all HIV+ patients were treated at a clinical centre with a high rate of retention in care [26].

The model considers two types of delay: *delayed diagnosis and health system organization delay*.

Delayed diagnosis is the time elapsed between the first false negative test performed and the TB diagnosis. We considered no delay in diagnosis if the first test performed is positive.

Health system organization delay is the time interval between TB diagnosis and treatment initiation, due to a delay in the delivery of results [27].

The main consequence of a delayed diagnosis is a delay in treatment initiation. It was assumed that on average HIV+ patients are seen at the clinical centre every 3 months (90 days), thus during the subsequent clinic visit TB would be identified and treated. The medical literature states [28, 29] that patients with active pulmonary TB are generally still infectious for 15 days after initiation of anti-TB treatment, although this assumption has been widely debated [30–32]. We considered patients to be infectious for 15 days after treatment initiation, as this period is still widely considered the infective phase for TB patients in treatment in most guidelines [33]. In addition, during the time elapsed between the first false-negative test and the second positive test, the patient would be infectious, so another important negative consequence is the increase in the number of newly transmitted TB infections. Total delay in diagnosis and treatment initiation for false negative patients considered was 105 days (90 days’ time between the two tests plus 15 days infectious period). Traditionally, the estimated risk of transmission for an active pulmonary TB case is 10 infections per year [34]. Although HIV infected patients with TB are less often contagious and present higher rates of SMEAR-negative TB [35, 36], scarce data are available regarding the number of transmission episodes per year for HIV/TB co-infected patients. Hence, we assumed a 50% lower value for HIV-positive patients, or 5 new transmitted cases per year. As this represents one of the most important parameters in the simulation, a range of 2–10 was considered in the sensitivity analysis.

The *health system organization delay* (health system delay for short) influence treatment initiation. In the scenario where this aspect was considered, we assumed a *health system delay* of 61 days, as estimated in a previous study in Mozambique [27].

Although there is a general consensus on the increase of mortality in TB/HIV patients due to delayed diagnosis and consequent delayed treatment, the magnitude of such increase could change according to multiple variables. According to the literature [37–39], we set mortality at 20%, but in the sensitivity analysis we varied the parameter between 10 to 30%.

### Cost analysis

The human resources cost required to administer the different tests were estimated from the actual cost of DREAM program. For cost of treatment (rifampicin 150 mg / isoniazid 75 mg / pyrazinamide 400 mg / ethambutol 275 mg for 4 months plus rifampicin 150 mg / isoniazid 75 mg for 2 months) the average cost incurred by the Global Fund for TB therapy of 9.44 USD was used [40]. For the cost of SMEAR, MTB/RIF and LAM test, we used the value reported in other studies [41–43].

All investment costs were discounted to the base year of the study, considering the average life of 5 years for machines. The economic burden for transmitted TB infections was set at 847 USD per infection as evaluated in a prior study [44]. A 3% discount rate was applied to annualized fixed costs. We also used a discount rate for future benefits of 3%, which is the usual routine in cost-effectiveness analysis (CEA). All other parameters utilized in the study are detailed in Table 2. We considered interventions to be very cost-effective if ICER was below the per capita Gross Domestic Product (GDP) of Mozambique (382 USD in 2016) and cost-effective if ICER was below three times the per capita GDP (1,146 USD) [45] as stated in the CEA guidelines of the WHO [46].

**Table 2:** Key parameters.

Parameter	Values	Sources
Median age sample, years	35	[10]
Risk of TB transmission (cases per year)	5.0	[34]
***Prevalence***		
TB among HIV patients, %	10.1	[10]
HIV patients with CD4 <200 /mm^3^, %	36.2	[DREAM data]
HIV/TB co-infected patients with CD4<200 /mm^3^, %	68.0	[DREAM data]
***Mortality***	-	-
In care (early treatment), %	5.0	Calculated
Among false negative (delayed treatment), %	20.0	Calculated
***Disability weights***		-
TB patients	0.399	[43]
AIDS (in care)	0.053	[43]
***Life expectancy***	-	-
Population in Mozambique, years	57.6	[13]
***Survival***	-	-
HIV+ and TB patient not treated for TB, years	0.665	[20]
HIV+ and TB treated, years*	12.9	[20,22,23]
***Costs of diagnostic***		
*4SS during first visit after HIV test*	-	-
Medical doctor (cost per test)	$4.00	[DREAM data]
**Total**	$4.00	-
*SMEAR*	-	-
Laboratory technician (cost per test)	$1.56	[43]
Microscopy	$1,500	[41]
Annualized cost (5 years life)	$327.53	Calculated
Maintenance (per year)	$19.15	Calculated
Cost microscopy (per test)	$0.04	Calculated
Consumables	$2.00	Calculated
**Total per test**	**$3.13**	
*MTB/RIF*	-	-
Laboratory technician (cost per test)	$1.10	[43]
MTB/RIF device	$17,000	[43]
Annualized cost (5 years life)	$1,992.92	[43]
Maintenance (per year)	$1,800.00	[43]
Cost of device per test	$3.64	[43]
Consumables	$9.98	[43]
**Total per test**	$14.72	[43]
*LAM*	**-**	
**Total per test**	**$3.99**	[42]
***Cost of TB treatment***		
Drugs	$9.44	[40]
Nurse (drug administration)	$0.40	[DREAM data]
**Total per patient**	**$9.84**	Calculated
**Economic burden of a new TB case due delayed diagnosis and treatment**	**$847.00**	[44]

*Note: patients treated for both HIV and TB.

### Sensitivity analysis

We performed a one-way sensitivity analysis on key parameters by varying each parameter over broad ranges of plausible values, supported by the literature whenever possible, and assessing the impact on results.

## Results

### Clinical impact and cost-effectiveness

In a cohort of 1,000 HIV+ patients in Mozambique, both the protocols using MTB/RIF and LF-LAM/ MTB/RIF generated 1,281 and 1,254 DALY’s saved respectively. The standard protocol showed a lower number of DALY’s than MTB/RIF and LF-LAM / MTB/RIF with a difference of 174 and 147 DALY’s due to the total number of treated TB-infected patients according to specificity and sensitivity of tests. In fact, the standard protocol diagnosed less 13 TB cases than other protocols (Table 3).

**Table 3:** Effectiveness of the three screening protocols.

	Sample size	Expected positive	Positive		Negative		Mortality		Total patients on therapy	No. of newly/additional transmitted TB infections due to delayed TB diagnosis and treatment in index case		DALY saved for treated patients
			*True*	*False*	*True*	*False*	*In care*	*False negative*		Delayed TB Diagnosis	Health systemDelay	
*Standard*	1000	101	34	0	899	67	5	13	83	96	70	1107
	%	(10.10%)	(3.40%)	(0%)	(89.9%)	(6.70%)	(0.50%)	(1.30%)				
*MTB/RIF*	1000	101	99	7	892	2	5	0	103	3	87	1281
		(10.10%)	(9.90%)	(0.07%)	(89.2%)	(0.20%)	(0.50%)	(0%)				
*LF-LAM / MTB/RIF*	1000	101	990%)	37	863	2	7	0	131	3	111	1254
		(10.10%)	(9.90%)	(3.7%)	(86.3%)	(0.20%)	(0.70%)	(0%)				

**Fig 2:**
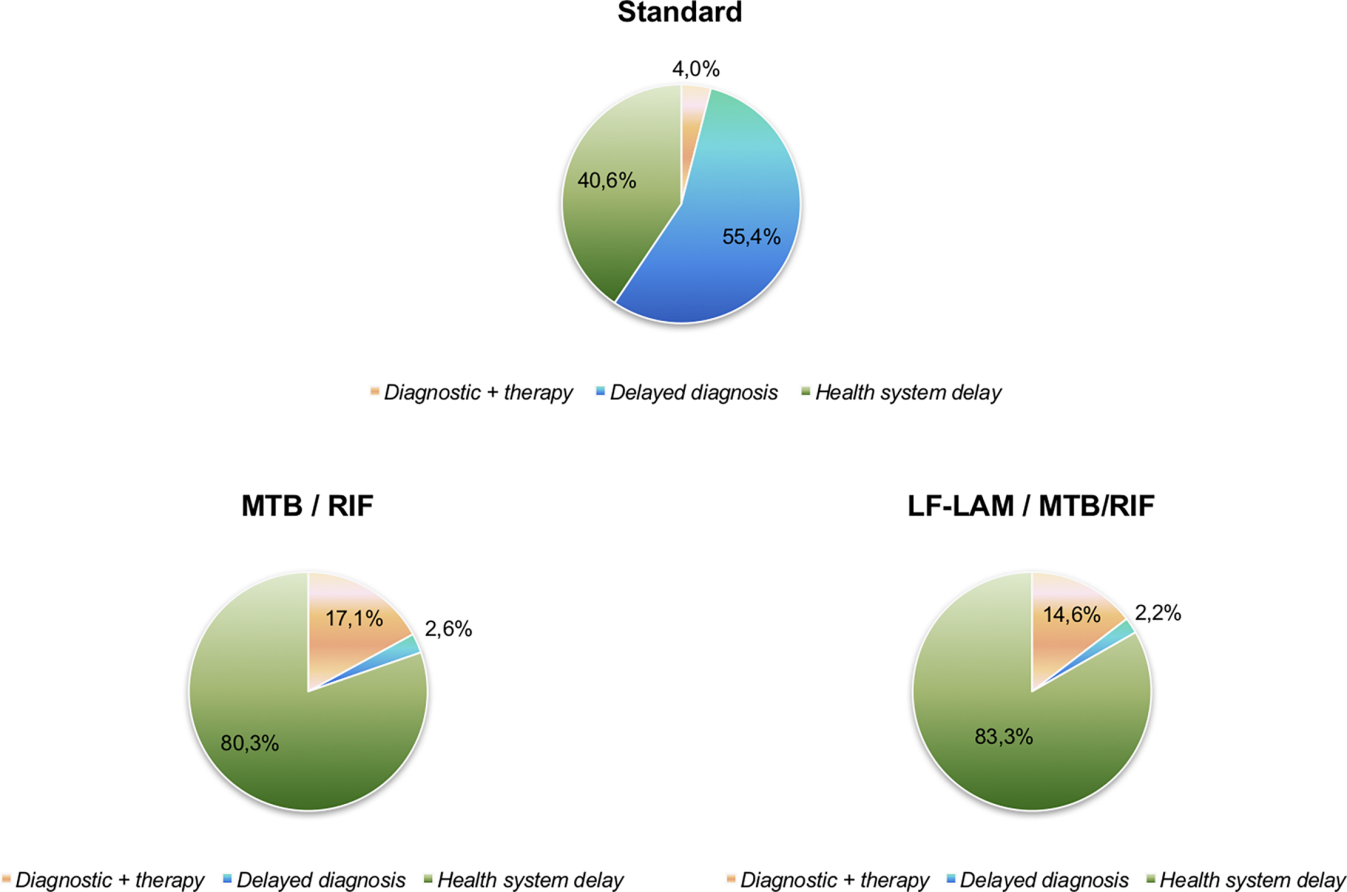
Itemized costs as percentage of total costs.

Overall, the MTB/RIF protocol was less costly as compared to the standard protocol (incremental cost $54,964 for 1,000 patients) and to the LF-LAM / MTB/RIF protocol (incremental cost $20,934 for 1,000 patients). As shown in Table 4, with the standard protocol, the higher cost item is the *cost of newly transmitted TB infections because of delayed diagnosis in the index case* (55.4% of the total cost). The cost of TB diagnostic and therapy in standard protocol is lower than MTB/RIF and LF-LAM / MTB/RIF protocols. Fig 2 shows the itemized costs as percentage of total costs. Approximately 40% of the total cost of the standard protocol is due to the cost of additional transmitted infections because of health system delay which represents approximately 80% of total cost in the other protocols. The cost per DALY saved including new TB infections in others due to delayed diagnosis and treatment initiation is around 5 fold higher in the standard protocol the MTB/RIF or LF-LAM / MTB/RIF, due to the higher cost of new infections and lower DALY saved. Because of delayed diagnosis and health system delay, the cost per DALY saved in the standard protocol is 1.4 and 1.8-fold that of the LF-LAM / MTB/RIF and MTB/RIF protocols, respectively.

**Table 4:** Total cost of the three protocols.

	4SS	SMEAR	MTB/RIF	LAM	Therapy	Cost of diagnostic + therapy (a)	Cost of newly transmitted infections because of delayed diagnosis (b)	Total direct costs (a) + (b)	Cost of additional transmitted infections because of health system delay (c)	Total direct and indirect costs (a)+(b)+(c)
	$									
*Standard*	4,000	1,076	-	-	817	5,893	81,625	87,519	59,708	**147,226**
*MTB/RIF*	-	-	14,717	-	1,014	15,731	2,437	18,168	74,095	**92,263**
*LF-LAM/ MTB/RIF*	-	-	13,790	1,442	1,290	16,522	2,437	18,959	94,237	**113,196**

The ICER per DALY saved using the MTB/RIF protocol versus the standard protocol excluding the cost of newly transmitted infections was also determined. In this scenario, the MTB/RIF protocol was still considered cost-effective as compared to the standard protocol with an ICER of 56.54 USD per DALY saved (Table 5). Given that the ICER of MTB/RIF and LF-LAM / MTB/RIF protocols versus standard protocol is lower than per capita GDP of Mozambique, the diagnosis and treatment evaluated are very cost-effective.

**Table 5:** Cost per DALY saved and ICER per DALY saved.

	Cost per DALY saved ($) including newly/additional transmitted TB infections due to delayed TB diagnosis and treatment		ICER per DALY saved ($/DALY)
	Delayed diagnosis	Delayed diagnosis + health system delay	
*Standard*	79.06	133.00	
*MTB/RIF*	14.18	72.02	56.54
*LF-LAM / MTB/RIF*	15.12	90.27	72.31

### Sensitivity analysis

We performed a one-way sensitivity analysis on the ICER for the MTB/RIF protocol versus the standard protocol and cost per DALY saved which included newly transmitted infections due to delayed diagnosis. We excluded the cost of additional transmitted infections because of the health system delay. Overall, the most influential parameters seem prevalence of TB in HIV+ patients followed by cost of MTB/RIF (Table 6). We depicted the most relevant parameters in two tornado diagrams (3–4).

**Table 6:** One-way sensitivity analysis.

Parameters				Cost per DALY saved ($)			ICER
				Standard	MTB/RIF	LF-LAM / MTB/RIF	MTB/RIF vs Standard
	Base case	Min	Max	Base case 79,06	Base case 14,18	Base case 15,12	Base case 56,54
Survival for HIV+ and TB+ patient not treated for TB, years	0,665	0,500	2,000	79,42–76,04	14,26–13,65	15,19–14,54	57,20–54,65
Risk of TB transmission (cases per year)	5,00	2,00	10,00	34,82–152,79	13,04–16,08	13,95–17,06	56,54–56,54
Prevalence of TB among HIV patients, %	10,10	5,05	15,15	85,61–78,86	26,28–11,04	28,49–10,13	123,18–36,82
Mortality In care (early treatment), %	5,00	2,50	7,50	77,19–82,0	13,92–14,62	14,54–15,59	56,87–56,54
Mortality Among false negative (delayed diagnosis and treatment), %	20,00	10,00	30,00	73,78–86,24	14,18–14,33	15,12–15,27	104,03–39,12
Discount rate	3,00	0,00	6,00	66,40–93,01	11,91–16,68	12,70–17,79	47,53–66,47
Survival for HIV+ and TB+ patients post treatment, years	12,9	6,450	19,350	66,40–93,01	11,91–16,68	12,70–17,79	47,53–66,47
Cost 4SS, **$**	4,00	2,00	6,00	134,64–60,73	24,19–10,90	25,76–11,62	97,40–43,53
Cost SMEAR (per test), **$**	3,13	1,56	4,69	77,25–80,87	14,18–14,18	15,12–15,12	68,03–45,04
Cost MTB/RIF (per test), **$**	14,72	7,36	22,08	79,06–79,06	8,44–19,93	9,62–20,62	14,25–98,83
Cost LF-LAM (per test), **$**	3,99	1,99	5,98	79,06–79,06	14,18–14,18	14,54–15,69	56,54–56,54
Cost TB therapy, **$**	9,84	4,92	14,77	78,69–79,43	13,79–14,58	14,60–15,63	55,97–57,10
Cost of newly transmitted TB infections, **$**	847,00	423,50	1.270,50	42,19–115,93	13,23–15,13	14,15–16,09	56,54–56,54
Days of delay in treatment for undiagnosed TB case	105	52,50	157,50	42,19–115,93	13,23–15,13	14,15–16,09	56,54–56,54

#### ICER per MTB/RIF protocol versus standard protocol

ICER was strongly influenced by the prevalence of TB in HIV+ patients. When prevalence included the minimum value (5.05%), the ICER reached the maximum value of 123.18 as opposed to the case base of 56.54. Cost-effectiveness was sensitive to the cost of the MTB/RIF assays with a minimum value of 14.25 if the cost was lower ($7.36) than the base case.

**Fig 3:**
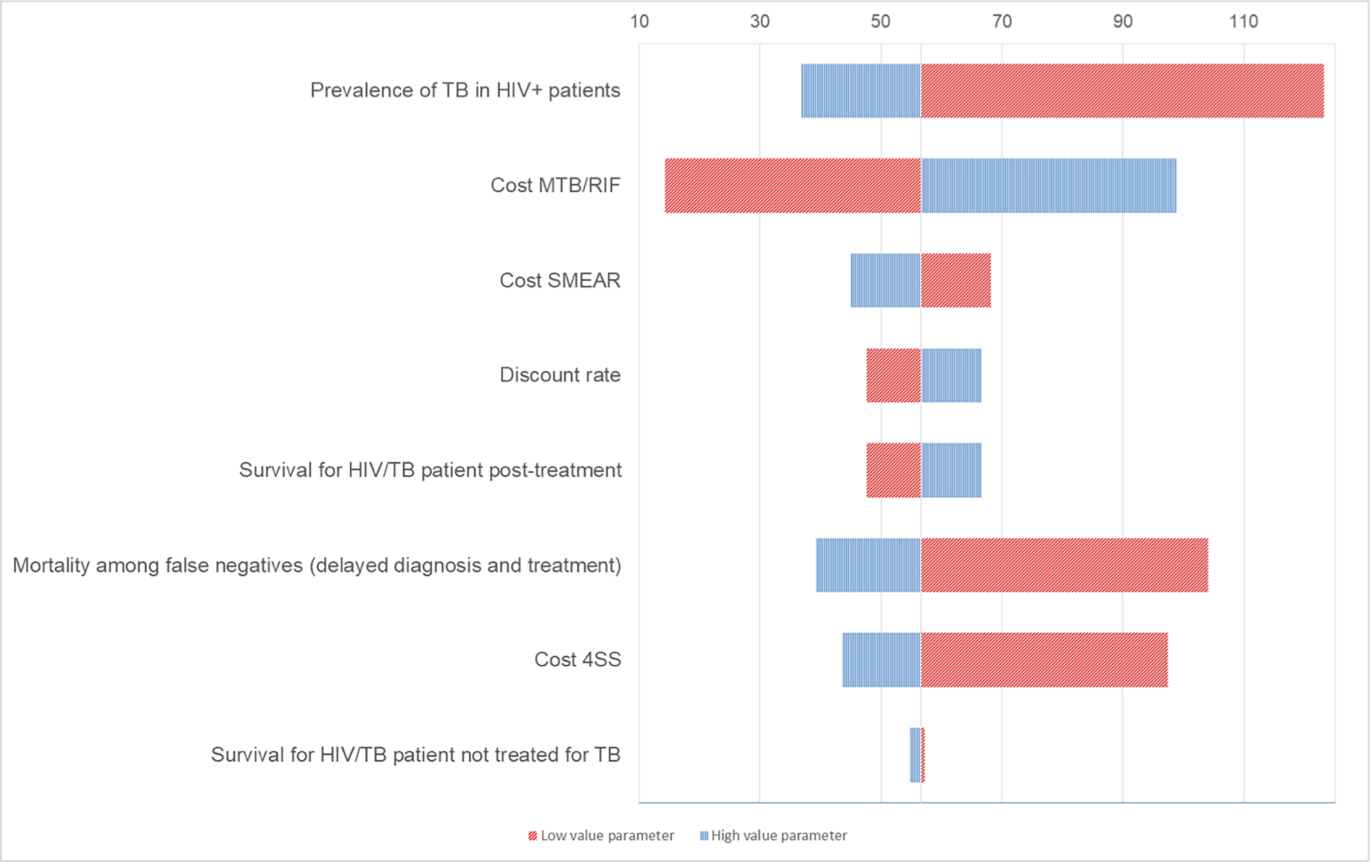
Tornado diagram. **One-way sensitivity analysis of ICER in MTB/RIF protocol versus Standard protocol (including newly TB transmitted infections due to delayed diagnosis).** Only parameters with greatest influence are shown. Vertical line is at base-case ICER of $56.54.

Mortality among the false negatives due to delayed diagnosis and cost of 4SS affected the ICER more than other parameters as cost of SMEAR, discount rate and survival of HIV/TB patients not treated for TB. However, all protocols remained cost-effective even when using the lowest parameters estimated (Fig 3).

#### Cost per DALY saved

**Fig 4:**
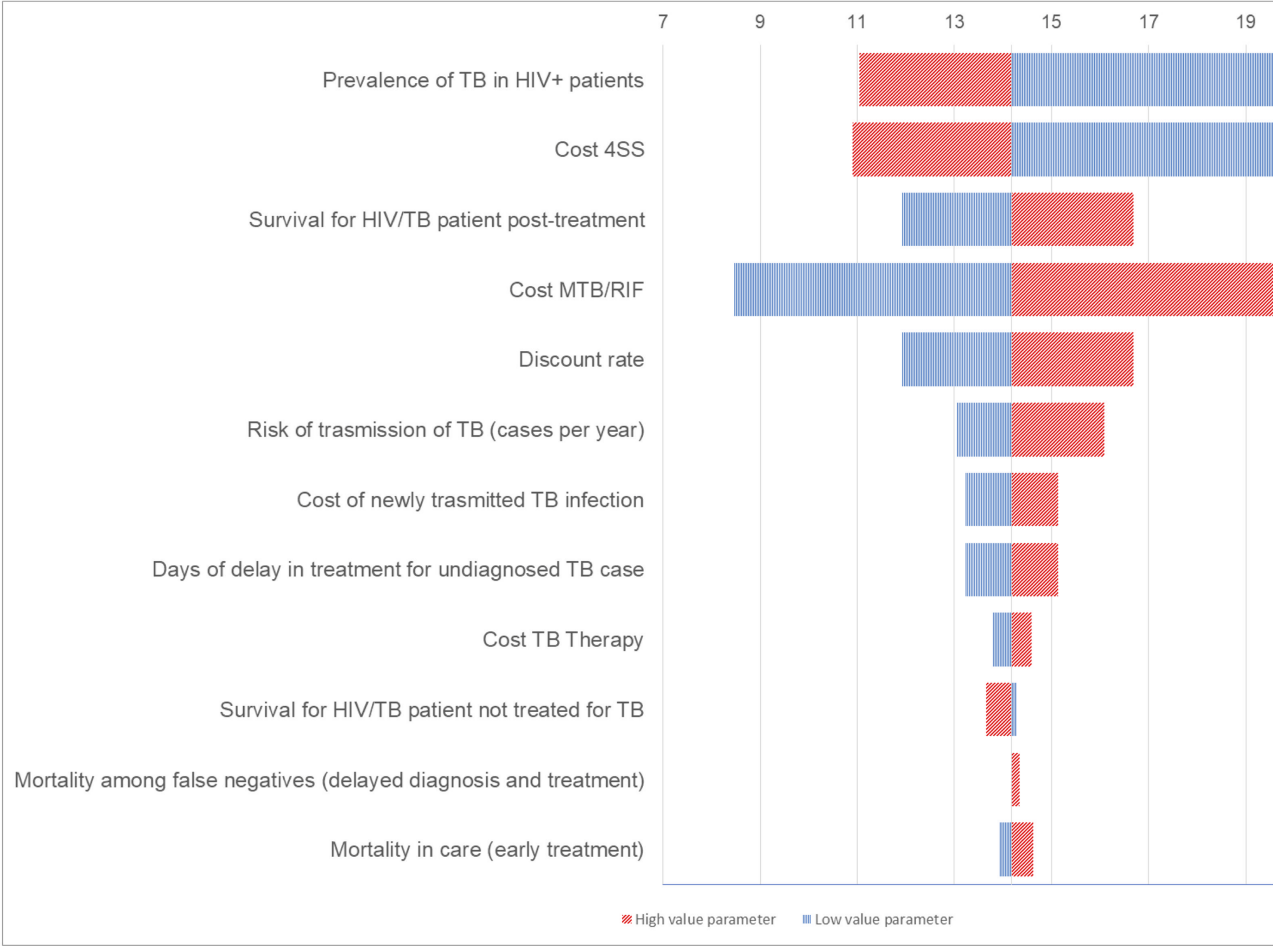
Tornado diagram. **One-way sensitivity analysis of cost per DALY saved (including newly TB transmitted infections due to delayed diagnosis) in MTB/RIF protocol.** Only parameters with the greatest influence are shown. Vertical line is at base-case cost per DALY saved of $14.18.

For the MTB/RIF protocol the cost per DALY saved (including newly transmitted TB infections in case of delayed diagnosis) was mainly influenced by the prevalence of TB, cost of 4SS, cost of MTB/RIF assay and survival of HIV and TB infected patients post- treatment (Fig 4).

## Discussion

MTB/RIF is already recommended for TB diagnosis in HIV+ suspected patients [4]. The use of MTB/RIF as a systematic screening test [12] in all HIV+ patients irrespectively of symptoms is not recommended at the moment, whereas 4SS is recommended for systematic TB screening in HIV+ patients.

Our study takes into consideration diagnostic tests that are currently available, and tries to identify the best way to use them in order to reduce costs from a public health point of view.

In this perspective, the most interesting result from the present study is the clear advantage of adopting MTB/RIF, also from the economic point of view, as a first step screening test on all HIV+ patients irrespectively of any symptom.

The protocol that is currently recommended in many African contexts (4SS followed by MTB/RIF in suspected patients) has not been included in the analysis. Previous study already demonstrated that the 4SS generates a high number of false negatives [10], which in turn generates an additional number of previously undiagnosed new TB infections, with a high cost to society.

To our knowledge, no CEAs comparing MTB/RIF, SMEAR and LF-LAM have yet been performed in Mozambique. If we consider other countries, the most relevant CEAs were conducted in Uganda [43], and in South Africa [37,47], plus a multi-country study (Uganda, India and South Africa) [21].

All the studies determined the cost-effectiveness and the ICER considering the cost per DALY saved, excluding Andrews et al. [37] that considers the year of life saved.

In three studies the use of MTB/RIF alone [21], MTB/RIF combined with LF-LAM [43] and MTB/RIF followed by MTB/RIF [37] were considered cost-effective compared to the other existing diagnostic protocols. Only the study performed by Vassall et al. [47] found MTB/RIF and SMEAR not showing difference in terms of cost-effectiveness.

No studies assessed the cost of newly/additional transmitted TB infections due to the delayed diagnosis and health system delay.

In Uganda, Shah et al. [43] considered the cost of diagnostic/therapeutic delays (i.e. diagnosed only on repeated presentation to care) assuming a relative increase in TB mortality in the analysis, whereas Andrews et al. [37] considered the delayed diagnosis on average two months due to false-negative results of SMEAR-negative or MTB/RIF-negative TB, and monthly mortality probability with untreated TB of 0.086. Vassall et al. [21] assumed that TB undiagnosed cases return to the clinic after 3 months, with 10% of undiagnosed cases becoming SMEAR positive within that time. Unlike Andrews et al. [37] and Shah et al. [43] who selected a cohort of HIV positive patients with TB symptoms, multi-drug resistance (MDR) or drug susceptible patients, Vassall et al. [21] considered a wider cohort of patients: new or previously treated, HIV-negative and HIV-positive and MDR or drug susceptible.

However, our different result may be influenced by several factors: 1) the *cohort of patients* that included only HIV positive patients needing long life care and frequent access to medical facilities; 2) *cost analysis* did not take into account follow-up and hospitalization costs as well as antibiotics and other treatments, MDR-TB treatments and chest radiographies; 3) the *cost of newly/additional transmitted TB infections because of delayed diagnosis and treatment*; 4) the *context* of Mozambique.

Our results did not underline clear preference for the use of LF-LAM to diagnose pulmonary TB in HIV+ patients with CD4 cells count <200/mm^3^, from a strictly economic point of view. In fact, the use of LF-LAM reduces the number of further tests needed to diagnose TB only for patients testing positive, while patients testing negative should undergo two tests (LF-LAM + MTB/RIF), resulting in increased costs.

One of the most important factors in this study was the increase in the frequency of newly transmitted cases of TB due to delay in diagnosis and treatment initiation and the economic burden of these infections to society. Not considering this element in the analysis, the MTB/RIF protocol was no longer strongly dominant, since the total cost of the traditional system was lower, but the ICER ratio, even in the sensitivity analysis, always remained very cost-effective.

### Limitations

This study didn’t take into consideration a possible intervention on the organizational model that could reduce time for treatment initiation. The ability to reduce the time between the patient's access to the clinical centre, diagnosis and subsequent treatment is a fundamental element determining the efficiency of a TB diagnostic system. This represents perhaps one of the most important challenges in the African context, especially in remote areas far from health services [27, 38].

As mentioned, all data about sensitivity and specificity of SMEAR, MTB/RIF and LF-LAM were derived from previous studies or international guidelines and not directly comparing tests and culture. This could be a limitation, although all the data used from other studies are based on sensitivity/specificity analysis comparing test with gold standard (generally culture). Moreover, our study is a simulation of different protocols based mainly on derived data, whereas an analysis that would take into account only direct data on the field comparing different tests (including culture) and economic aspects could have a wider impact.

The study did not consider, among the consequences of undiagnosed cases, the possibility that such patients are no longer intercepted by the healthcare system, since it has been assumed that PLHIV needs frequent visits. Furthermore, the consequences of delayed diagnosis and treatment initiation on the efficacy of the therapy have not been taken into consideration. These two assumptions reduced the negative impact of a false-negative TB diagnosis. However, removing these assumption, would reinforce the conclusions of the study on the need to adopt more sensitive screening methods.

As far as the cost analysis is concerned, it has been maintained within the time frame of a single year, so no extra costs or savings in the future have been taken into consideration for the different diagnostic protocols and for human and instrumental resources.

The need for more effective, although more expensive, TB diagnosis and treatment, reinforced by the present study, is mainly based on the very high TB burden in many societies. However, the value of this burden is very variable. The value used in the present analysis assessed in the Tanimura study [44] of 847 USD has a very high variability depending on the context. In fact, the same Tanimura [44] indicates a range of 55–8,198 USD. Moreover, a large part of this cost is due to the loss of income of individuals affected by TB, especially among the poorest.

The results of the study are applicable above all to those countries where the mechanisms of social care and protection are weaker or non-existent, or where the level of poverty is very high, while they may not be valid in those countries where the welfare state is more efficient or in the countries with medium-high income.

At the same time, the impact of education and awareness-raising actions to increase the demand for these services by the patients themselves, who are often poorly informed about TB and therefore access services late in the course of illness, should be taken into account when planning health policies.

## Conclusions

This study showed that MTB/RIF protocol is cost-effective compared to standard and LF-LAM / MTB/RIF protocol in PLHIV, especially in countries with high HIV and TB prevalence such as Mozambique.

This is reinforced by the fact that in Mozambique the incidence of TB is increasing, in contrast with the overall global trend, so it is important to adopt any strategy that can reduce the number of new cases, and certainly it includes early diagnosis. Conversely, the disadvantages of the adoption of MTB/RIF as a screening protocol in all HIV+ patients irrespectively of any TB symptom remain: a) the higher cost of the device, b) the cost of maintenance and durability, and c) the relatively greater difficulty in its use. However, with regard to points a) and b), a wider adoption of MTB/RIF could lead to a reduction in costs and a greater ability to maintain the methodology due to economies of scale. This is especially true in countries like Mozambique where the high prevalence of TB reduces the risk of underutilization of machines, especially in the urban and peri-urban context. We must also take into account the relative availability of economic resources due to the presence of major international donors in this area, such as the Global Fund against HIV, TB and Malaria, and the US Presidential Emergency Plan for AIDS Relief. For the same reasons, it is feasible, and not particularly expensive, to support training for professional figures.

## Supporting information

S1 File Model for the analisys with input and output data. (XLSX)
